# Influenza Organism

**Published:** 1895-06

**Authors:** 


The Influenza Organisms.—That epidemic influenza is to be classed among the germ diseases will be doubted only by those who hold that there is no such thing as a germ disease at all. It matters little by way of proof whether the specific germ has been discovered as yet or not, although there is reasonable probability that the bacilli described by Pfeiffer and Kitasato are the sought-for cause.—*Northwestern Lancet.*

